# Severe Acute Respiratory Syndrome Coronavirus 2 Prevalence, Seroprevalence, and Exposure among Evacuees from Wuhan, China, 2020

**DOI:** 10.3201/eid2609.201590

**Published:** 2020-09

**Authors:** Benjamin D. Hallowell, Christina M. Carlson, Jesica R. Jacobs, Mary Pomeroy, Jonathan Steinberg, Mark W. Tenforde, Emily McDonald, Loretta Foster, Leora R. Feldstein, Melissa A. Rolfes, Amber Haynes, Glen R. Abedi, George S. Odongo, Kim Saruwatari, Errin C. Rider, Gina Douville, Neenaben Bhakta, Panagiotis Maniatis, Stephen Lindstrom, Natalie J. Thornburg, Xiaoyan Lu, Brett L. Whitaker, Shifaq Kamili, Senthilkumar K. Sakthivel, Lijuan Wang, Lakshmi Malapati, Janna R. Murray, Brian Lynch, Martin Cetron, Clive Brown, Shahrokh Roohi, Lisa Rotz, Denise Borntrager, Kenta Ishii, Kathleen Moser, Mohammad Rasheed, Brandi Freeman, Sandra Lester, Kizzmekia S. Corbett, Olubukola M. Abiona, Geoffrey B. Hutchinson, Barney S. Graham, Nicki Pesik, Barbara Mahon, Christopher Braden, Casey Barton Behravesh, Rebekah Stewart, Nancy Knight, Aron J. Hall, Marie E. Killerby

**Affiliations:** Centers for Disease Control and Prevention, Atlanta, Georgia, USA (B.D. Hallowell, C.M. Carlson, J.R. Jacobs, M. Pomeroy, J. Steinberg, M.W. Tenforde, E. McDonald, L. Foster, L.R. Feldstein, M.A. Rolfes, A. Haynes, G.R. Abedi, G.S. Odongo, P. Maniatis, S. Lindstrom, N.J. Thornburg, X. Lu, B.L. Whitaker, S. Kamili, S.K. Sakthivel, L. Wang, L. Malapati, J.R. Murray, B. Lynch, M. Cetron, C. Brown, S. Roohi, L. Rotz, D. Borntrager, K. Ishii, K. Moser, B. Freeman, N. Pesik, B. Mahon, C. Braden, C. Barton Behravesh, R. Stewart, N. Knight, A.J. Hall, M.E. Killerby);; Riverside University Health System-Public Health, Riverside, California, USA (K. Saruwatari, E.C. Rider, G. Douville, N. Bhakta);; Synergy America Inc., Duluth, Georgia, USA (M. Rasheed, S. Lester);; National Institutes of Health, Bethesda, Maryland, USA (K.S. Corbett, O.M. Abiona, G.B. Hutchinson, B.S. Graham); 1 Current affiliation: Department of Health, Providence, Rhode Island, USA

**Keywords:** 2019 novel coronavirus disease, coronavirus disease, COVID-19, severe acute respiratory syndrome coronavirus 2, SARS-CoV-2, viruses, respiratory infections, zoonoses, quarantine, Wuhan, China, evacuees

## Abstract

To determine prevalence of, seroprevalence of, and potential exposure to severe acute respiratory syndrome coronavirus 2 (SARS-CoV-2) among a cohort of evacuees returning to the United States from Wuhan, China, in January 2020, we conducted a cross-sectional study of quarantined evacuees from 1 repatriation flight. Overall, 193 of 195 evacuees completed exposure surveys and submitted upper respiratory or serum specimens or both at arrival in the United States. Nearly all evacuees had taken preventive measures to limit potential exposure while in Wuhan, and none had detectable SARS-CoV-2 in upper respiratory tract specimens, suggesting the absence of asymptomatic respiratory shedding among this group at the time of testing. Evidence of antibodies to SARS-CoV-2 was detected in 1 evacuee, who reported experiencing no symptoms or high-risk exposures in the previous 2 months. These findings demonstrated that this group of evacuees posed a low risk of introducing SARS-CoV-2 to the United States.

On December 31, 2019, a cluster of severe pneumonia cases in Wuhan, Hubei Province, China, was reported (*1*). On January 7, 2020, a novel coronavirus, severe acute respiratory syndrome coronavirus 2 (SARS-CoV-2), was isolated from samples associated with the cluster (*2*,*3*). As of May 1, 2020, a total of 3,175,207 coronavirus disease (COVID-19) cases had been confirmed and 224,172 persons had died worldwide; 84,385 cases and 4,643 deaths were in China (*4*). Also as of May 1, 2020, the US Centers for Disease Control and Prevention (CDC) was reporting ongoing worldwide transmission (*5*).

On January 20, 2020, a case of coronavirus disease (COVID-19) was confirmed in a US patient who had recently traveled to Wuhan (*6*). To slow the spread of the epidemic, on January 23, the government of China enacted a travel ban restricting all travel into and out of Wuhan, including air and rail travel, and suspending operation of buses, subways, and ferries within the city (*7*). As of January 23, a total of 571 confirmed COVID-19 cases had been reported in China (*8*).

After China enacted the travel ban, the US Department of State planned evacuation flights for US citizens and other third country nationals in Wuhan. We describe the demographic and clinical characteristics, potential exposures to SARS-CoV-2, personal protective measures implemented, and SARS-CoV-2 real-time reverse transcription PCR (rRT-PCR) and serologic test results for evacuees from 1 repatriation flight from Wuhan. These data can be used to better determine SARS-CoV-2 epidemiology, including assessing the point prevalence of past and current SARS-CoV-2 infections in this cohort and identifying factors associated with infection in this cohort. These findings can also be used to help estimate the initial risk for transmission to contacts in the United States posed by evacuees from Wuhan and are relevant to current and future implementation of public health control measures, such as isolation and quarantine.

## Methods

We investigated quarantined evacuees from a January 28, 2020, repatriation flight from Wuhan to the United States. Before the flight departed Wuhan, evacuees were evaluated to ensure that they had no fever or respiratory signs/symptoms. At arrival in the United States and again at the quarantine facility, evacuees were asked to complete a US Traveler’s Health Declaration form disclosing any symptoms; they were also screened for illness and fever, asked about symptoms in the past 72 hours, and asked about any high-risk exposures (including working in or visiting healthcare settings; caring for or visiting persons with fever, respiratory illness, or a confirmed COVID-19 diagnosis; or visiting any live animal markets) in Wuhan in the past 14 days. Those who reported symptoms or high-risk exposures were evaluated by a CDC Quarantine Medical Officer, who determined if they required further evaluation and isolation from the quarantined cohort.

Nasopharyngeal and oropharyngeal swab samples and serum specimens were obtained from participating evacuees when they arrived at the quarantine station in the United States. As part of quarantine procedures, evacuees were actively monitored for fever and respiratory signs/symptoms for 14 days after departure from Wuhan; any evacuee in whom either fever or respiratory signs/symptoms developed during this time was evaluated for COVID-19 (*9*), and additional nasopharyngeal and oropharyngeal specimens were collected (*10*,*11*). All specimens were collected, processed, and shipped to CDC for testing (*10*,*11*). Presence of SARS-CoV-2 in nasopharyngeal and oropharyngeal swab samples was confirmed by rRT-PCR detection of viral RNA in respiratory specimens (*12*). Serum specimens were initially tested for SARS-CoV-2 antibodies by SARS-CoV-2 ELISA (Appendix 1).

We asked evacuees to complete a detailed, self-administered survey during the flight from Wuhan (Appendix 2). The survey captured information on demographics, clinical signs/symptoms, travel outside of Hubei Province, face mask use, limitation of time spent in public, and past high-risk exposures (including contact with confirmed COVID-19 case-patients; persons with fever, acute respiratory illness, or both; healthcare and laboratory facilities; and animals and live animal markets). We assessed high-risk exposures over the past 2 weeks and the past 2 months. We compared high-risk exposures over the past 2 weeks with rRT-PCR results for persons who provided an upper respiratory specimen (because 14 days was the upper end of the estimated incubation period for COVID-19 [*13**,**14*]). We also compared high-risk exposures over the past 2 months with the serologic test results for evacuees who provided a serum sample (because SARS-CoV-2 had probably been circulating for the 2 months before their departure [*15*]).

We entered survey responses into REDCap electronic data capture tools hosted at CDC (*16*), and all entries were verified by a second reviewer for accuracy and completeness. Data were analyzed by using SAS software version 9.4 (SAS Institute, Inc., https://www.sas.com).

CDC determined that this investigation was public health surveillance (US Department of Health and Human Services, Title 45 Code of Federal Regulations 46, Protection of Human Subjects). Evacuees’ participation in the collection of biological specimens and the survey was voluntary.

## Results

At the time of arrival in the United States, no evacuee had a measured fever or reported any signs or symptoms that required further evaluation. Of the 195 evacuees, 193 completed surveys; 99% (191/193) of respondents provided a nasopharyngeal sample, an oropharyngeal sample, or 1 of each for SARS-CoV-2 rRT-PCR testing, and 96% (186/193) provided a serum sample for testing. The median age of all 193 evacuees was 42 (range 0–74) years, and 53% (100/189) were male (Table 1). Most were either Asian (49%, 94/192) or non-Hispanic White (35%, 68/192).

**Table 1:** Demographic characteristics of 193 evacuees on a repatriation flight from Wuhan, China, to the United States, January 2020

Characteristic	No./total no. (%)*
Age group, y	
<18	32/193 (17)
18–44	68/193 (35)
45–64	83/193 (43)
>65	10/193 (5)
Sex	
M	100/189 (53)
F	89/189 (47)
Race/ethnicity	
White	68/192 (35)
Black	6/192 (3)
Asian	94/192 (49)
Multiracial	13/192 (7)
Hispanic	11/192 (6)
Underlying medical condition	
Chronic lung disease	1/191 (1)
Asthma/reactive airway disease	7/191 (4)
Diabetes mellitus type 1	2/187 (1)
Diabetes mellitus type 2	4/191 (2)
Hypertension	14/192 (7)
Chronic heart or cardiovascular disease	0/191 (0)
Chronic kidney disease	1/191 (1)
Liver disease	1/191 (1)
Noncancer immunosuppressive condition	1/189 (1)
Neurologic/neurodevelopmental disorder	1/191 (1)
Other chronic disease	11/191 (6)
Specimen submitted	
Nasopharyngeal and/or oropharyngeal swab sample	191/193 (99)
Serum	186/193 (96)

*Data for persons for whom responses were missing were excluded from the numerator and denominator.

One evacuee reported having had close contact with a person with laboratory-confirmed COVID-19 in the previous 2 weeks. Specifically, reported exposures included direct physical contact, being within 6 feet of the person while that person was coughing or sneezing, taking an object handed from or handled by the person, and traveling in the same vehicle as the person (Table 2). No other evacuees reported exposure to a person with laboratory-confirmed COVID-19 in the previous 2 months. However, 6% (12/191) reported having had close contact with a person with fever, acute respiratory illness, or both in the previous 2 weeks and 16% (30/186) in the previous 2 months (Table 2). One evacuee had visited a live animal market in the previous 2 weeks and 5% (9/186) in the previous 2 months. Three percent (6/191) of evacuees had visited settings with nondomesticated live animals in the previous 2 weeks and 5% (10/186) in the previous 2 months. One percent (2/191) of evacuees had had direct physical contact with a nondomesticated live animal (both instances with stray dogs) in the previous 2 weeks. No additional evacuees had had direct physical contact with a nondomesticated live animal in the previous 2 months. 

**Table 2:** Potential exposures to severe acute respiratory syndrome coronavirus 2 by 193 evacuees returning from Wuhan, China, to the United States, January 2020*

Exposure risk factors	No./total no. (%)†
Relevant exposures for serology results‡	
Animal contact	
Visited the Huanan Seafood Market in past 2 mo	1/186 (1)
Visited any live animal market in past 2 mo	9/186 (5)
Visited any settings with domesticated animals in past 2 mo	39/186 (21)
Visited any settings with nondomesticated animals in past 2 mo	8/186 (4)
Had direct contact with any animals in past 2 mo	52/186 (28)
Human contact	
Had close contact with laboratory-confirmed COVID-19 case-patient in past 2 mo	1/186 (1)
Had close contact with person with fever and/or acute respiratory illness in past 2 mo	30/186 (16)
High-risk settings	
Visited a healthcare setting (not in United States) in past *2* mo	8/186 (4)
Worked in a healthcare setting in Wuhan in past *2* mo	0/186 (0)
Worked in a laboratory setting in Wuhan in past *2* mo	0/186 (0)
Travel	
Did not travel outside of Hubei Province, China, in past *2* mo	62/186 (33)
Relevant exposures for PCR results§	
Animal contact	
Visited any live animal market in past 2 wk	1/191 (1)
Visited any settings with domesticated animals in past 2 wk	15/191 (8)
Visited any settings with nondomesticated animals in past 2 wk	6/191 (3)
Had direct physical contact with live domestic animals in past 2 wk	36/191 (19)
Had direct physical contact with live nondomestic animals in past 2 wk	2/191 (1)
Human contact	
Had close contact with laboratory-confirmed COVID-19 case-patient in past 2 wk	1/191 (1)
Had close contact with person with fever and/or acute respiratory illness in past 2 wk	12/191 (6)
High-risk settings	
Visited a healthcare setting (not in United States) in past 2 wk	7/191 (4)

*COVID-19, coronavirus disease. †Data for persons for whom responses were missing were excluded from the denominator. ‡Limited to exposures within the past 2 mo and to persons who submitted serum sample. §Limited to exposures within the past 2 wk and to persons who submitted a nasopharyngeal and/or oropharyngeal swab specimen.

During the previous month, after hearing about COVID-19 cases in Wuhan, 95% (178/188) of evacuees reported having limited their time in public in Wuhan, including avoiding public gatherings (87%), public transportation (84%), and all public settings (e.g., grocery stores or restaurants; 70%) (Table 3). In addition, in the previous month, after hearing about COVID-19 cases in Wuhan, 76% of evacuees reported having worn a face mask while in public spaces. This finding represented a significant increase from the 34% of evacuees who reported having worn a face mask while in public spaces in the previous 2 months (McNemar test statistic 74.05; p<0.0001).

**Table 3:** Precautions taken to prevent infection with severe acute respiratory syndrome coronavirus 2 by 193 evacuees while in Wuhan, China*

Precaution	No./total no. (%)†		
	Total, n = 193	Submitted NP or OP sample,‡ n = 191	Submitted serum sample,§ n = 186
Face mask use			
Usually wore a face mask in past *2* mo while in public	63/185 (34)	63/184 (34)	63/178 (35)
Usually wore a face mask in past 1 mo while in public after hearing about COVID-19	143/188 (76)	143/187 (76)	138/181 (76)
Limited time in public			
In past 1 mo after hearing about COVID-19	178/188 (95)	176/186 (95)	171/181 (94)
By taking the following precautions			
Avoided public transport	150/178 (84)	148/176 (84)	144/171 (84)
Avoided public gatherings	154/178 (87)	153/176 (87)	148/171 (87)
Did not attend work¶	53/123 (43)	53/121 (44)	53/123 (43)
Did not attend school/university#	19/30 (63)	19/30 (63)	15/25 (60)
Avoided all public settings (e.g., grocery stores, restaurants)	125/178 (70)	124/176 (70)	119/171 (70)

*COVID-19, coronavirus disease; NP, nasopharyngeal swab; OP, oropharyngeal swab. †Data for persons for whom responses were missing were excluded from the denominator. ‡Limited to persons who submitted an NP and/or OP specimen. ^§^Limited to persons who submitted serum specimen. ¶Limited to persons who reported an occupation (other than student, stay-at-home parent, or retired). #Limited to persons 2–18 years of age and those reporting student as occupation.

Five percent (9/193) of evacuees reported having experienced signs or symptoms associated with COVID-19 (measured or subjective fever, cough, shortness of breath) in the previous 2 weeks, and 12% (24/193) reported signs/symptoms associated with COVID-19 in the previous 2 months. One evacuee who reported signs/symptoms associated with COVID-19 in the previous 2 weeks sought medical care, and no evacuee required hospitalization while in Wuhan (Table 4).

**Table 4:** Signs/symptoms, clinical course, and past medical history for evacuees reporting illness who were on a repatriation flight from Wuhan, China, to the United States in early 2020*

Characteristic	No./total no. (%)†	
	Self-reported illness in past 2 mo, n = 39	Self-reported illness in past 2 wk, n = 13
Sign/symptom		
Measured fever	5/36 (14)	3/12 (25)
Subjective fever	16/37 (43)	2/13 (15)
Cough	15/36 (42)	6/12 (50)
Sore throat	21/38 (55)	9/13 (69)
Muscle aches	10/37 (27)	2/11 (18)
Headache	12/37 (32)	1/12 (8)
Shortness of breath	2/34 (6)	1/12 (8)
Vomiting	3/33 (9)	1/11 (9)
Diarrhea	7/36 (19)	1/12 (8)
Fatigue	16/37 (43)	3/12 (25)
Other	10/30 (33)	6/10 (60)
Any coronavirus sign/symptom‡	24/39 (62)	9/13 (69)
Identified as a person under investigation for COVID-19 signs/symptoms§	10/39 (26)	2/13 (15)
Sought medical care for illness in past 2 wk	1/39 (3)	0/13 (0)

*All persons who self-reported illness submitted serum and a nasopharyngeal or oropharyngeal swab specimen. COVID-19, coronavirus disease. †Persons for whom responses were missing were excluded from the numerator and denominator. ‡Measured fever OR subjective fever, cough, or shortness of breath. §Measured or subjective fever AND shortness of breath or cough.

SARS-CoV-2 was not detected by rRT-PCR in any of the 190 nasopharyngeal or 190 oropharyngeal swab specimens collected from 191 unique evacuees (189 provided nasopharyngeal and oropharyngeal samples, 1 nasopharyngeal sample only, and 1 oropharyngeal sample only). During the 14-day quarantine period, fever developed in 2 evacuees; additional nasopharyngeal and oropharyngeal swab specimens were collected and tested, and SARS-CoV-2 was not detected in either specimen type.

One evacuee showed serologic evidence of a past SARS-CoV-2 infection. Serum from that person had antibodies against SARS-CoV-2 at titers of 400 determined by ELISA and 320 determined by microneutralization test. This person was male, was in the 19-44–year age group, was traveling without any family members, and reported no signs/symptoms associated with COVID-19 in the past 2 months. He reported no high-risk exposures (including exposure to or contact with live animals, live animal markets, persons known to be ill with COVID-19, or persons with fever or acute respiratory signs/symptoms). He reported that since early January he had spent limited time out in public, including avoiding public transport, avoiding public gatherings, and not attending school/university. ELISA results for the remaining 185 serum specimens measured SARS-CoV-2 antibody titers at <400, and the samples were therefore considered seronegative.

## Discussion

Our report on SARS-CoV-2 prevalence, seroprevalence, and potential exposures among evacuees returning from Wuhan is part of the public health response enacted to slow transmission of SARS-CoV-2 in the United States. Although this population of evacuees is probably not representative of all Wuhan residents in terms of risk of acquiring SARS-CoV-2 infection, our results indicate limited exposure to SARS-CoV-2 among this group of early evacuees from Wuhan.

Compared with previously reported COVID-19 case-patients in Wuhan, our population was younger (median 42 vs. 59 years of age) and their reported frequency of potential SARS-CoV-2 exposures was lower, including exposure to persons with respiratory signs/symptoms, work-associated healthcare exposures, and exposure to live animal markets (*15*). Of note, although our questionnaire covered exposure to animals and animal markets, most transmission within Wuhan during the evacuees’ relevant exposure period before the repatriation flight to the United States was probably human-to-human (*15**,**17*). Our study population, which consisted predominantly of US expatriates, probably had other factors that reduced their risk for exposure and were not documented as part of our investigation. For example, it is possible that the expatriates’ households in Wuhan were smaller than other households in Wuhan, which has been associated with a lower risk for transmission (*18*–*21*); however, because we did not document household size in our investigation, we cannot show such an association. Nearly all evacuees took preventive measures to limit potential exposure to SARS-CoV-2 while in Wuhan. However, 16% of evacuees did have direct contact with persons who had fever or acute respiratory illness.

Previous investigations among evacuees traveling from Wuhan to Germany and Japan detected SARS-CoV-2 RNA in 7 asymptomatic persons (*22**,**23*), suggesting that symptom-based screening alone may not be effective for detecting SARS-CoV-2 infection. Evacuees in our study underwent intensive screening such that no evacuee had signs/symptoms at the time of evacuation and none had detectable SARS-CoV-2 in upper respiratory tract specimens, suggesting the absence of asymptomatic respiratory shedding among this group at the time of testing. In addition, no SARS-CoV-2 was detected in respiratory specimens from the 2 evacuees in whom fever developed during quarantine. The lack of SARS-CoV-2 detection in asymptomatic travelers at the time of testing and in the 2 travelers in whom fever developed could result from a lower risk for exposure among this group compared with Wuhan residents or other reported evacuees (*22**,**23*).

The ELISA and microneutralization tests used in this investigation have produced robust responses to serum from confirmed SARS-CoV-2 patients (B. Freeman et al., unpub data, https://www.biorxiv.org/content/10.1101/2020.04.24.057323v2 28). Although 24 evacuees reported signs/symptoms associated with COVID-19 (subjective fever, cough, or shortness of breath) in the previous 2 months, none were seropositive for SARS-CoV-2. In contrast, an antibody response was detected in 1 person who did not report illness in the previous 2 months, indicating past SARS-CoV-2 infection, suggestive of past asymptomatic or mildly symptomatic infection. The overall seroprevalence of 1% suggests a low level of exposure to SARS-CoV-2 over the preceding 2 months in Wuhan. However, a lack of antibody response may not mean an absence of past infection; serologic responses are not always detected in persons with SARS-CoV-2 infection (*24*). 

Efforts by this cohort to limit their exposure by limiting their time in public may have helped prevent infection, even in a city with extensive ongoing community transmission. Because SARS-CoV-2 seems to be transmitted primarily through respiratory droplets, limiting time in public may have helped prevent infection because proximity to infected persons is needed for virus transmission (*25*). Before the evacuees in our study departed Wuhan, China was implementing measures to control SARS-CoV-2 by suspending public transport and vehicle traffic and canceling Lunar New Year gatherings (*7*). CDC currently recommends that all persons wear cloth face coverings in public; the purpose is to help protect others from potential droplet exposure, not to protect the persons wearing the face coverings (*26*). Thus, although 76% of evacuees reported mask use after hearing about COVID-19 in Wuhan, the effect of individual mask use on their individual risk of acquiring infection is unknown..

Information about virus prevalence, seroprevalence, and possible SARS-CoV-2 exposures in this population of evacuees has the potential to inform current and future quarantine and isolation policies. In this population, who underwent intensive screening and monitoring, we detected no evidence of current infection with SARS-CoV-2 and very limited evidence of past infection. Other than the 193 evacuees included in our study, 3 cases of COVID-19 were detected in the United States during quarantine of later cohorts of evacuees after signs/symptoms developed and the evacuees underwent testing, demonstrating the value of quarantine and active monitoring of evacuees to detect COVID-19 cases (*27*).

Our investigation has limitations. First, the survey was self-administered and based on self-report; therefore, questions were open to interpretation and subject to reporting bias. Because respiratory specimens from asymptomatic persons were collected at a single point in time, we are unable to show whether asymptomatic shedding might have occurred later during quarantine. Also, rRT-PCR assays and serologic tests are inherently limited by their individual sensitivity and specificity; however, we believe that the limitations of test specificity and sensitivity across this population of evacuees were minimal. In addition, because serum samples were collected only once, at the time of US arrival, we were unable to detect antibodies that may have developed later.

As of May 1, a total of 1,062,446 COVID-19 cases had been confirmed in the United States, including 39 in repatriated persons (3 cases in 808 returned evacuees across 5 flights from Hubei Province and 36 cases from the Diamond Princess cruise ship) (*28*). Initial efforts to slow introduction of SARS-CoV-2 to the United States began in January 2020 and included quarantine of persons with high-risk exposures, screening of travelers at airports, and isolation and contact tracing of confirmed case-patients (*28*). Our investigation demonstrated that this group of evacuees posed a low risk of introducing SARS-CoV-2 to the United States, and their exposure to SARS-CoV-2 in Wuhan was probably limited. These results should help inform public health guidance on quarantine and isolation measures for travelers arriving from high-risk areas and further characterize the epidemiology of this emerging virus.

Appendix 1 Supplemental methods for study of severe acute respiratory syndrome coronavirus 2 prevalence, seroprevalence, and exposure among evacuees from Wuhan, China, 2020.

Appendix 2 Questionnaire used in study of severe acute respiratory syndrome coronavirus 2 prevalence, seroprevalence, and exposure among evacuees from Wuhan, China, 2020.
