# Supplementary material for: Severe Acute Respiratory Syndrome Coronavirus 2 Prevalence, Seroprevalence, and Exposure among Evacuees from Wuhan, China, 2020
Source: Emerg Infect Dis. 2020 Sep;26(9):1998–2004. doi: 10.3201/eid2609.201590 (PMC7454104; doi:10.3201/eid2609.201590)
Supplement: Appendix 1 — Supplemental methods for study of severe acute respiratory syndrome coronavirus 2 prevalence, seroprevalence, and exposure among evacuees from Wuhan, China, 2020. [file 20-1590-Techapp-s1.pdf]

Article DOI: https://doi.org/10.3201/eid2609.201590

Severe Acute Respiratory Syndrome Coronavirus 2 Prevalence, Seroprevalence, and Exposure Among Evacuees from Wuhan, China, 2020

Appendix 1

Supplemental Methods

Serum specimens were initially tested for anti–SARS-CoV-2 antibodies by SARS-CoV-2 ELISA using recombinantly expressed viral spike protein S1/S2 (*1*). Briefly, plates were coated overnight at 4°C with a concentration of 150 ng/ml of S1/S2. All serum specimens from evacuees were then added to plates in serial 4-fold dilutions from 1:100 to 1:6,400 and washed, followed by the addition of horseradish peroxidase (HRP-conjugated anti human IgM/IgA/IgG (SeraCare Life Sciences, Milford, MA) with 2,2’-azino-di (3- ethylbenzthiazoline- 6-sulfonate; ABTS) peroxidase substrate system to detect binding and to determine endpoint antibody titers (SeraCareLife Sciences, Milford, MA). Absorbance was read at 405 nm and 490 nm as background correction. The highest dilution with a background correction optical density >0.4 was the endpoint titer. Any specimens with titers ≥400 were considered positive by ELISA. Serum samples that were positive by ELISA were confirmed by microneutralization test using live SARS-CoV-2 USA_WA1_2020 in a Biosafety Level 3 laboratory, using the MERS-CoV microneutralization workflow (*2*).

References

1. Wrapp D, Wang N, Corbett KS, Goldsmith JA, Hsieh C-L, Abiona O, et al. Cryo-EM structure of the 2019-nCoV spike in the prefusion conformation. Science. 2020;367:1260–3. PubMed https://doi.org/10.1126/science.abb2507

2. Trivedi S, Miao C, Al-Abdallat MM, Haddadin A, Alqasrawi S, Iblan I, et al. Inclusion of MERS-spike protein ELISA in algorithm to determine serologic evidence of MERS-CoV infection. J Med Virol. 2018;90:367–71. PubMed https://doi.org/10.1002/jmv.24948
