# Supplementary material for: Severe Acute Respiratory Syndrome Coronavirus 2 Prevalence, Seroprevalence, and Exposure among Evacuees from Wuhan, China, 2020
Source: Emerg Infect Dis. 2020 Sep;26(9):1998–2004. doi: 10.3201/eid2609.201590 (PMC7454104; doi:10.3201/eid2609.201590)
Supplement: Appendix 2 — Questionnaire used in study of severe acute respiratory syndrome coronavirus 2 prevalence, seroprevalence, and exposure among evacuees from Wuhan, China, 2020. [file 20-1590-Techapp-s2.pdf]

Article DOI: https://doi.org/10.3201/eid2609.201590

Severe Acute Respiratory Syndrome Coronavirus 2 Prevalence, Seroprevalence, and Exposure Among Evacuees from Wuhan, China, 2020

Appendix 2

Evacuee Survey

The following pages contain the self-administered survey given to 95 evacuees to be completed during a repatriation flight from Wuhan, China, to the United States on January 28, 2020. Of the 95 evacuees, 93 completed the survey.

Form Approved: OMB: 0920-1011 Exp. 4/23/2020

Specimen ID
(for CDC use only)

2019-nCoV Wuhan Exposure Questionnaire

CDC ID (*CDC use only*): ☐

Please complete this form for each person traveling in your group.

Today's date: ____/____/_____ (MM/DD/YYYY)

Demographic Information

1. Age (years): ____________ Age in months (If aged less than 1 year): ________________
2. Ethnicity: ☐ Hispanic/Latino ☐ Non-Hispanic/Latino
3. Race: (check all that apply) ☐ White ☐ Asian ☐ American Indian/Alaska Native ☐ Black ☐ Native Hawaiian/Other Pacific Islander
4. Sex: ☐ Male ☐ Female
5. Occupation: ____
6. County of Destination:________________________ State of Destination:________
   Residency: ☐ US resident ☐ Non-US resident
   If non US resident, nationality: _______________________________

Symptoms and healthcare

7. Have you been sick in the past 2 months? ☐ Yes ☐ No (skip to Q.15) ☐ Unknown (skip to Q.15)
   If yes, what date did the symptoms associated with this illness start? _____/_____/_______ (MM/DD/YYYY)
8. During this illness, did you experience any of the following symptoms?

Symptom	Symptom Present?	Symptom	Symptom Present?
Measured Fever (highest temp _________ ºF)	☐ Yes ☐ No ☐ Unknown	Vomiting	☐ Yes ☐ No ☐ Unknown
Subjective fever (felt feverish)	☐ Yes ☐ No ☐ Unknown	Diarrhea	☐ Yes ☐ No ☐ Unknown
Cough	☐ Yes ☐ No ☐ Unknown	Eye infection/redness	☐ Yes ☐ No ☐ Unknown
Sore Throat	☐ Yes ☐ No ☐ Unknown	Rash	☐ Yes ☐ No ☐ Unknown
Muscle aches	☐ Yes ☐ No ☐ Unknown	Fatigue	☐ Yes ☐ No ☐ Unknown
Headache	☐ Yes ☐ No ☐ Unknown	Seizures	☐ Yes ☐ No ☐ Unknown
Shortness of breath	☐ Yes ☐ No ☐ Unknown	Other, specify below:	☐ Yes ☐ No ☐ Unknown

   Other symptoms:____
   ____

9. Are you feeling back to normal? ☐ Yes ☐ No
   If yes, when did you feel back to normal? _____/_____/_____ (MM/DD/YYYY)
10. Did you receive any medical care for the illness?
   ☐ Yes ☐ No (skip to Q.15) ☐ Unknown (skip to Q.15)
11. Where and on which date did you seek care first after this illness started (check all that apply)?
   ☐ Outpatient **Date**:_____/_____/_____ (MM/DD/YYYY) ☐ Emergency room **Date**:_____/_____/_____ (MM/DD/YYYY)
   ☐ Retail store clinic **Date**:_____/_____/_____ (MM/DD/YYYY) ☐ Health department **Date**:_____/_____/_____ (MM/DD/YYYY)
   ☐ Urgent care **Date**:_____/_____/_____ (MM/DD/YYYY) ☐ Other __________ **Date**:_____/_____/_____ (MM/DD/YYYY)
   ☐ Unknown
12. Were you hospitalized for the illness? ☐ Yes ☐ No (skip to Q.21) ☐ Unknown (skip to Q.21)
13. Date of hospital admission? ______/_____/_______(MM/DD/YYYY) Date of hospital discharge? ______/_____/_______ (MM/DD/YYYY)
14. Have you been sick a second time the past 2 months? ☐ Yes ☐ No ☐ Unknown
    If yes, fill out additional questions found in the appendix.

....

Person completing this form. First name: ______________________________ Last name:__________________________

If this form is being completed for someone else, please enter that person's name below.

Specimen ID
(for CDC use only)

First name: ______________________________ Last name:__________________________

CDC ID (*CDC use only*): ☐

Public reporting burden of this collection of information is estimated to average 30 minutes per response, including the time for reviewing instructions, searching existing data sources, gathering and maintaining the data needed, and completing and reviewing the collection of information. An agency may not conduct or sponsor, and a person is not required to respond to a collection of information unless it displays a currently valid OMB control number. Send comments regarding this burden estimate or any other aspect of this collection of information including suggestions for reducing this burden to CDC/ATSDR Reports Clearance Officer; 1600 Clifton Road NE, MS D-74 Atlanta, Georgia 30333; ATTN: PRA (0920-1011)

Past Medical History

15. Do you have any of the following chronic medical conditions? Please specify **ALL** conditions that qualify.

Condition	Response			If YES, specify
Chronic lung diseases	☐Yes	☐No	☐Unknown	
Asthma/reactive airway disease	☐Yes	☐No	☐Unknown	
Other chronic lung disease	☐Yes	☐No	☐Unknown	
Diabetes Mellitus				
Diabetes Mellitus Type 1	☐Yes	☐No	☐Unknown	
Diabetes Mellitus Type 2	☐Yes	☐No	☐Unknown	
Hypertension	☐Yes	☐No	☐Unknown	
Chronic heart or cardiovascular disease	☐Yes	☐No	☐Unknown	
Chronic kidney disease	☐Yes	☐No	☐Unknown	
Liver disease	☐Yes	☐No	☐Unknown	
Non-cancer immunosuppressive condition or therapy	☐Yes	☐No	☐Unknown	
Cancer chemotherapy in past 12 months	☐Yes	☐No	☐Unknown	
Neurologic/neurodevelopmental disorder	☐Yes	☐No	☐Unknown	
Other chronic diseases	☐Yes	☐No	☐Unknown	

16. (Female only) Are you pregnant?
☐ Yes (weeks pregnant) ___________ ☐ No ☐ Unknown
17. Do you currently smoke?
☐ Yes ☐ No ☐ Unknown
    a. If yes, how many packs per day? _________________
    b. For how many years? ________________
18. Do you currently vape or use e-cigarettes?
☐ Yes ☐ No ☐ Unknown

Travel history

19. In the last 2 months did you travel outside of Wuhan, Hubei Province, China?
☐ Yes ☐ No ☐ Unknown
20. Where did you travel in the last 2 months (list **ALL** locations, including overnight transits)?

Trip 1: Departure date: _____/_____/_____ (MM/DD/YYYY) Departure city/country: _______________
Arrival date: _____/_____/_____ (MM/DD/YYYY) Arrival city/country: _______________
Trip 2: Departure date: _____/_____/_____ (MM/DD/YYYY) Departure city/country: _______________
Arrival date: _____/_____/_____ (MM/DD/YYYY) Arrival city/country: _______________
Trip 3: Departure date: _____/_____/_____ (MM/DD/YYYY) Departure city/country: _______________
Arrival date: _____/_____/_____ (MM/DD/YYYY) Arrival city/country: _______________

(If extra travel dates describe in comments section at end)

Time in Wuhan

The following questions all refer to exposures during your time in Wuhan, China. Please know that some questions ask about the last 2 months, and some ask about the last 2 weeks.

21. In the last **2 months**, did you visit Huanan Seafood Market? ☐ Yes ☐ No ☐ Unknown
22. In the last **2 months,** did you visit any other live animal markets? ☐ Yes ☐ No ☐ Unknown
23. If visited in the last **2 weeks**, dates: MM/DD/YYYY____
24. In the last **2 months**, did you purchase any products at live animal markets? ☐ Yes ☐ No ☐ Unknown
25. If so, list: ____
____
26. In the last **2 months**, did you visit any other settings whether at home or away from home where live animals were present, including livestock, pets, or wildlife? ☐ Yes ☐ No ☐ Unknown
If yes, describe setting:____
27. If visited in the last **2 weeks,** dates: MM/DD/YYYY____

28. In the past **2 months**, did you have any direct contact (such as touching or holding) with any type of animal including livestock, pets, or wildlife whether at home or away from home? (list **ALL** animal exposures including pets)?
☐ Yes ☐ No ☐ Unknown

29. If you contacted animals in the last **2 weeks,** please list here:

City/Country contact(s) occurred	Type of animal contacted (livestock, pets, wildlife,)	Date(s) / date range of contact (in the past **2 WEEKS**) (MM/DD/YYYY - MM/DD/YYYY)	Contact setting(s) **(check all that apply)**
		____/____/____ - ____/____/____	☐ Home ☐ Work ☐ Farm ☐ Animal Market ☐ Zoo ☐Other____________
		____/____/____ - ____/____/____	☐ Home ☐ Work ☐ Farm ☐ Animal Market ☐ Zoo ☐Other____________
		____/____/____ - ____/____/____	☐ Home ☐ Work ☐ Farm ☐ Animal Market ☐ Zoo ☐Other____________
		____/____/____ - ____/____/____	☐ Home ☐ Work ☐ Farm ☐ Animal Market ☐ Zoo ☐Other____________
		____/____/____ - ____/____/____	☐ Home ☐ Work ☐ Farm ☐ Animal Market ☐ Zoo ☐Other____________
If additional exposures, please include in the appendix			

30. In the last **2 months**, did you have close contact with a person diagnosed with 2019-nCoV during the time that the person was sick?
☐ Yes ☐ No ☐ Unknown

If contact was in the last 2 weeks, dates: MM/DD/YYYY____

31. When you were exposed to the person diagnosed with 2019-nCoV, while that person was sick, did you:

Exposure to Confirmed 2019-nCoV patient				
		Date / Date range (MM/DD/YYYY - MM/DD/YYYY)	Estimated frequency (e.g. daily, 2x daily)	Estimated Duration (e.g. minutes, hours)
Have face to face contact?	☐Yes ☐No ☐Unknown	____/____/____ - ____/____/____		
Have direct physical contact? (e.g. hug, shake hands, etc.)	☐Yes ☐No ☐Unknown	____/____/____ - ____/____/____		
Physically within 6 feet of the case?	☐Yes ☐No ☐Unknown	____/____/____ - ____/____/____		
In close proximity (within 6 feet) while the case was coughing or sneezing?	☐Yes ☐No ☐Unknown	____/____/____ - ____/____/____		
Take an object handed from or handheld by the case? (e.g. pen, paper, fork, etc.)	☐Yes ☐No ☐Unknown	____/____/____ - ____/____/____		
In the same room as the case?	☐Yes ☐No ☐Unknown	____/____/____ - ____/____/____		
Travel in the same vehicle (car, bus, airplane), sitting within 6 feet of the case?	☐Yes ☐No ☐Unknown	____/____/____ - ____/____/____		
Live in the same house or apartment as a case	☐Yes ☐No ☐Unknown	____/____/____ - ____/____/____		

32. In the last **2 months**, did you have close contact with a person who had a fever and/or acute respiratory illness?

☐ Yes ☐ No ☐ Unknown

If contact was in the last **2 weeks**, dates: MM/DD/YYYY____

33. When you were exposed to someone with fever and/or acute respiratory illness, did you:

Exposure to person with fever and/or acute respiratory illness				
		Date / Date range (MM/DD/YYYY - MM/DD/YYYY)	Estimated frequency (e.g. daily, 2x daily)	Estimated Duration (e.g. minutes, hours)
Have face to face contact?	☐Yes ☐No ☐Unknown	_____/_____/____ - _____/_____/______		
Have direct physical contact? (e.g. hug, shake hands, etc.)	☐Yes ☐No ☐Unknown	_____/_____/____ - _____/_____/______		
Physically within 6 feet of the case?	☐Yes ☐No ☐Unknown	_____/_____/____ - _____/_____/______		
In close proximity (within 6 feet) while the case was coughing or sneezing?	☐Yes ☐No ☐Unknown	_____/_____/____ - _____/_____/______		
Take an object handed from or handheld by the case? (e.g. pen, paper, fork, etc.)	☐Yes ☐No ☐Unknown	_____/_____/____ - _____/_____/______		
In the same room as the case?	☐Yes ☐No ☐Unknown	_____/_____/____ - _____/_____/______		
Travel in the same vehicle (car, bus, airplane), sitting within 6 feet of the case?	☐Yes ☐No ☐Unknown	_____/_____/____ - _____/_____/______		
Live in the same house or apartment as a case	☐Yes ☐No ☐Unknown	_____/_____/____ - _____/_____/______		

34. In the last **2 months**, did you visit a healthcare setting? ☐ Yes ☐ No ☐ Unknown

If visit was in the last **2 weeks**, please list dates: MM/DD/YYYY____

If yes to any above, specify location, type of facility (nursing home, hospital, outpatient clinic, etc.): ____________________________

If yes to any above, did you have direct contact with other patients? ☐ Yes ☐ No ☐ Unknown

35. In the last **2 months,** did you work in a healthcare setting? ☐ Yes ☐ No *(Skip to Q38)* ☐ Unknown *(Skip to Q38)*

If yes to any above, specify location, type of facility (nursing home, hospital, outpatient clinic, etc.): ____________________________

If yes, in the last **2 weeks** give dates: (MM/DD/YYYY) ____

36. In the last **2 months,** did you care for a lab-confirmed 2019-nCoV patient(s) *(in a healthcare setting)*?
☐ Yes ☐ No ☐ Unknown

If yes, in the last **2 weeks** give dates: (MM/DD/YYYY) ____

When you were caring for a lab-confirmed 2019-nCoV patient(s):

	Lab-confirmed 2019-nCoV patient		
Were you within 6 feet of a confirmed case around the time they were positive?	☐ Yes	☐ No	☐ Unknown
For more than 10 minutes?	☐ Yes	☐ No	☐ Unknown
Which of the following PPE did you use while caring for the patient?			
Gloves	☐Never	☐Sometimes	☐Always
Gown	☐Never	☐Sometimes	☐Always
Surgical Mask	☐Never	☐Sometimes	☐Always
N95 mask or PAPR	☐Never	☐Sometimes	☐Always
Faceshield or goggles	☐Never	☐Sometimes	☐Always
Were you in the same room during aerosolizing procedures (including intubation, extubation, bronchoscopy, Code/CPR, open suctioning of airways, sputum induction)?	☐ Yes	☐ No	☐ Unknown

37. In the last **2 months,** did you care for a patient with fever and/or acute respiratory illness *(in a healthcare setting)*?
☐ Yes ☐ No ☐ Unknown Case

If yes, in the last **2 weeks** give dates: (MM/DD/YYYY) ____

When you were caring for a patient with fever and/or acute respiratory illness:

	Patient with fever and/or acute respiratory illness		
Were you within 6 feet of the patient around the time they were sick?	☐ Yes	☐ No	☐ Unknown
For more than 10 minutes?	☐ Yes	☐ No	☐ Unknown
Which of the following PPE did you use while caring for the patient?			
Gloves	☐Never	☐Sometimes	☐Always
Gown	☐Never	☐Sometimes	☐Always
Surgical Mask	☐Never	☐Sometimes	☐Always
N95 mask or PAPR	☐Never	☐Sometimes	☐Always
Faceshield or goggles	☐Never	☐Sometimes	☐Always
Were you in the same room during aerosolizing procedures (including intubation, extubation, bronchoscopy, Code/CPR, open suctioning of airways, sputum induction)?	☐ Yes	☐ No	☐ Unknown

38. In the last **2 months**, did you work in a laboratory setting handling blood, blood products, tissues or samples, or viral or bacterial samples?

    ☐ Yes ☐ No ☐ Unknown

    If yes, in the last **2 weeks** give dates: (MM/DD/YYYY) ____

    If yes, specify location and materials:____

39. In the last **2 months**, did you usually wear a face-mask while out in public? ☐ Yes ☐ No ☐ Unknown
40. In the last **1 mon**, after hearing about 2019-nCoV, did you usually wear a face-mask while out in public?

    ☐ Yes ☐ No ☐ Unknown

41. In the last **1 month**, after hearing about 2019-nCoV, did you limit time out in public? ☐ Yes ☐ No ☐ Unknown

    During what dates did you limit time out in public _____/_____/_____ (MM/DD/YYYY) to _____/_____/_____ (MM/DD/YYYY)

42. If yes, how did you limit time out in public (check all that apply):

    ☐ Avoid public transport ☐ Avoid public gatherings ☐ Not attend work ☐ Not attend school/university

    ☐ Avoiding all public settings including grocery stores, restaurants etc.

2019-nCoV Wuhan Exposure Investigation Form

Comments:

Appendix

Symptoms and healthcare for the Second Illness

1. Have you been sick a second time the past 2 months? ☐ Yes ☐ No ☐ Unknown
   If yes, complete the questions below.
2. What date did the symptoms associated with this illness start? _____/_____/_______ (MM/DD/YYYY)
3. During this illness, did you experience any of the following symptoms?

Symptom	Symptom Present?	Symptom	Symptom Present?
Measured Fever (highest temp _______ ºF)	☐ Yes ☐ No ☐ Unknown	Vomiting	☐ Yes ☐ No ☐ Unknown
Subjective fever (felt feverish)	☐ Yes ☐ No ☐ Unknown	Diarrhea	☐ Yes ☐ No ☐ Unknown
Cough	☐ Yes ☐ No ☐ Unknown	Eye infection/redness	☐ Yes ☐ No ☐ Unknown
Sore Throat	☐ Yes ☐ No ☐ Unknown	Rash	☐ Yes ☐ No ☐ Unknown
Muscle aches	☐ Yes ☐ No ☐ Unknown	Fatigue	☐ Yes ☐ No ☐ Unknown
Headache	☐ Yes ☐ No ☐ Unknown	Seizures	☐ Yes ☐ No ☐ Unknown
Shortness of breath	☐ Yes ☐ No ☐ Unknown	Other, specify below:	☐ Yes ☐ No ☐ Unknown

   Other symptoms:____

   ____

4. Are you feeling back to normal? ☐ Yes ☐ No (Note, if still experiencing symptoms, report as PUI)
   If yes, when did you feel back to normal? _____/_____/_____ (MM/DD/YYYY)
5. Did you receive any medical care for the illness?
   ☐ Yes ☐ No ☐ Unknown
6. Where and on what date did you seek care first after this illness started (check all that apply)?
7. Where and on what date did you seek care first after this illness started (check all that apply)?

☐ Outpatient **Date**:_____/_____/_____ (MM/DD/YYYY) ☐ Emergency room **Date**:_____/_____/_____ (MM/DD/YYYY)
☐ Retail store clinic **Date**:_____/_____/_____ (MM/DD/YYYY) ☐ Health department **Date**:_____/_____/_____ (MM/DD/YYYY)
☐ Urgent care **Date**:_____/_____/_____ (MM/DD/YYYY) ☐ Other __________ **Date**:_____/_____/_____ (MM/DD/YYYY)
☐ Unknown

8. Were you hospitalized for the illness? ☐ Yes ☐ No ☐ Unknown
Date of hospital admission? ______/_____/_______(MM/DD/YYYY) Date of hospital discharge? ______/_____/_______ (MM/DD/YYYY)

Additional Animal Exposures

City/Country contact(s) occurred	Type of animal contacted (wildlife, livestock, pets)	Date(s) / date range contact occurred (if one day ever date twice) (MM/DD/YYYY - MM/DD/YYYY)	Contact setting(s) (check all that apply)
		____/____/____ - ____/____/____	☐ Home ☐ Work ☐ Farm ☐ Animal Market ☐ Zoo ☐Other_____________
		____/____/____ - ____/____/____	☐ Home ☐ Work ☐ Farm ☐ Animal Market ☐ Zoo ☐Other_____________
		____/____/____ - ____/____/____	☐ Home ☐ Work ☐ Farm ☐ Animal Market ☐ Zoo ☐Other_____________
		____/____/____ - ____/____/____	☐ Home ☐ Work ☐ Farm ☐ Animal Market ☐ Zoo ☐Other_____________
		____/____/____ - ____/____/____	☐ Home ☐ Work ☐ Farm ☐ Animal Market ☐ Zoo ☐Other_____________
		____/____/____ - ____/____/____	☐ Home ☐ Work ☐ Farm ☐ Animal Market ☐ Zoo ☐Other_____________
		____/____/____ - ____/____/____	☐ Home ☐ Work ☐ Farm ☐ Animal Market ☐ Zoo ☐Other_____________
		____/____/____ - ____/____/____	☐ Home ☐ Work ☐ Farm ☐ Animal Market ☐ Zoo ☐Other_____________
		____/____/____ - ____/____/____	☐ Home ☐ Work ☐ Farm ☐ Animal Market ☐ Zoo ☐Other_____________
